# Detection of biofilms on explanted urinary incontinence slings and their relationship with pain, exposure, or urinary tract complications

**DOI:** 10.1128/aem.00735-26

**Published:** 2026-08-13

**Authors:** Nazish Abbas, Thomas Willmott, Paul M. Campbell, Gurdeep Singh, Maya Basu, Fiona Reid, Cecile El Chami, Andrew J. McBain

**Affiliations:** 1 Division of Developmental Biology & Medicine, School of Medical Sciences, Faculty of Biology, Medicine and Health, University of Manchester 5292, Manchester, United Kingdom; 2 Manchester Academic Health Science Centre, Warrell Unit, Saint Mary's Hospital, Manchester University NHS Foundation Trust 5293, Manchester, United Kingdom; 3 Division of Pharmacy and Optometry, School of Health Sciences, Faculty of Biology, Medicine and Health, University of Manchester 5292, Manchester, United Kingdom; 4 Lydia Becker Institute of Immunology and Inflammation, Manchester Academic Health Science Centre, Faculty of Biology, Medicine and Health, University of Manchester 5292, Manchester, United Kingdom; The University of Tennessee Knoxville, Knoxville, Tennessee, USA

**Keywords:** implant-associated infection, fluorescence *in situ* hybridisation, confocal microscopy, biofilms

## Abstract

Midurethral slings (MUS) are synthetic mesh implants used to treat stress urinary incontinence in women. Positioned via retropubic or transobturator approaches, they support the mid-urethra to maintain closure during increases in intra-abdominal pressure. Although generally effective, long-term MUS complications have been associated with chronic inflammation, pain, and infection, with explanted slings often showing bacterial colonization. The underlying mechanisms remain unclear, particularly the role of microbial biofilms. We examined the presence and characteristics of biofilms on MUS removed for various complications to better inform clinical management. MUS samples (*n* = 112) from 52 women were imaged using confocal laser scanning microscopy following application of a eubacterial FISH probe, TOTO-1, and DAPI. Biofilms were detected on all samples, forming dense bacterial aggregates along mesh fibers. The vaginal portions of the MUS demonstrated higher biofilm volumes than retropubic or obturator regions. However, mesh exposed through vaginal tissue did not show increased bacterial load. When all samples were analyzed, no significant differences in biofilm volume were observed between complication types, including chronic pain, vaginal or lower urinary tract perforation, and recurrent incontinence. While biofilms were universally present, greater volumes in the vaginal region did not correlate with worse clinical outcomes. These findings suggest a threshold between benign colonization and clinically significant infection. Variation in bacterial composition, rather than biofilm quantity, may influence host response and the development of mesh-related complications.

**IMPORTANCE:**

Biofilms are common on explanted midurethral slings, but their volume does not predict complications. These findings suggest that bacterial composition and host response, rather than bacterial load, drive mesh-related morbidity. Understanding these factors may improve mesh design and guide strategies to prevent infection and chronic complications.

## INTRODUCTION

The midurethral sling (MUS) was previously considered the gold-standard treatment for stress urinary incontinence (SUI) (1). MUS devices are usually made from polypropylene mesh (PPM), a material originally developed for abdominal hernia repair. The mesh used is typically monofilament and macroporous (Type 1 polypropylene mesh, characterized by pore sizes greater than 75 µm) (1), allowing the passage of immune cells through the pores to facilitate bacterial clearance (2, 3) and promote host tissue integration.

The MUS consists of a narrow polypropylene tape placed beneath the mid-urethra via a vaginal incision and passed either behind the pubic bone (retropubically) (4) or laterally through the obturator foramen (4, 5). These minimally invasive procedures became widely adopted because they offered shorter operative times and faster recovery than traditional non-mesh continence procedures, while maintaining high reported efficacy, with some studies reporting cure or success rates of approximately 80% or greater (6). However, increasing recognition of mesh related adverse events (7) led to national safety reviews and, in some countries, pauses or restrictions on the use of vaginally inserted mesh for stress urinary incontinence (7). Reported complications include chronic pain, dyspareunia, vaginal mesh exposure, voiding dysfunction, infection, and lower urinary tract perforation or erosion (7). The factors that determine why complications develop in some women but not others remain incompletely understood (8).

It is well established that synthetic implants provoke a foreign body reaction following the initial acute inflammatory response (9). This reaction is thought to persist for the lifespan of the implant. Animal and human studies indicate that polypropylene results in a higher inflammatory response when implanted vaginally, compared to abdominally (10–13). It has been demonstrated that vaginal surgery does not occur within a sterile field, despite routine surgical preparation measures to reduce the risk of infection (14); consequently, vaginally implanted materials are exposed to a non-sterile environment at the time of insertion. The current literature suggests that bacterial colonization of mesh may contribute to chronic inflammation and the development of complications (15). Explanted mesh generally yields positive bacterial culture results (16–18), and intraoperative swabs of the mesh during implantation show bacterial presence (19); both findings support the theory that chronic colonization plays a role in the development of complications.

Most mesh complications occur in the absence of overt infection, and many studies reporting bacterial presence on mesh describe only low bacterial burdens. It is therefore possible that bacteria colonize MUS at the time of insertion and persist throughout the lifespan of the implant. Type 1 polypropylene mesh is sufficiently macroporous to permit immune-cell infiltration, which would be expected to facilitate bacterial clearance and limit chronic colonization. However, bacteria may produce extracellular polymeric substances (EPS) forming biofilms, which may evade immune clearance. The role of biofilms in chronic inflammation and persistent infection has become widely accepted (20).

Biofilms have been shown to form on polypropylene mesh *in vitro* (21) and observed on hernia mesh removed for evidence of infection (22). A previous study, however, assessed biofilms on vaginally implanted prolapse mesh from only two women (23), one with a vaginal mesh exposure and one with overt infection. We have recently demonstrated that polypropylene midurethral sling mesh harbors a distinct microbiome that may be associated with mesh complications (24).

The current study assessed the presence and distribution of biofilms on midurethral slings (MUS). This required developing a methodology to visualize biofilms on explanted MUS from women with and without complications. A secondary aim was to quantify biofilm volume and evaluate its association with different types of mesh-related complications.

## MATERIALS AND METHODS

Women undergoing excision of their MUS between September 2020 and October 2022 at one specialist mesh center were recruited into this study. Women entered one of the following four groups depending on their indication for surgery: (i) chronic pain, i.e., women who attributed pelvic, vaginal, or groin pain to their mesh and were undergoing excision for this as the only reason; (ii) mesh exposure, i.e., women with mesh exposed in the vagina; (iii) lower urinary tract (LUT) perforation, i.e., women with mesh within the urethra or bladder; and (iv) control group, i.e., women undergoing surgery to excise mesh for any other reason, such as secondary incontinence surgery. A total of 122 samples of mesh were imaged for this study. Explanted MUS samples were stored in formalin in the Biobank until imaging.

### Validation of imaging

Validation of imaging methodology required the use of explanted MUS and MUS, which had never been implanted. MUS, which had never been implanted, served as a negative control (no biofilms) or was inoculated with lab strain *Pseudomonas aeruginosa* (PA01) to form a positive control. The purpose of this control was methodological validation rather than replication of the polymicrobial communities likely to be present on explanted clinical material.

### Preparation of control MUS

PPM MUS, which had never been implanted, was sterilized in an ethanol bath. The MUS was then cut into 1 × 1 cm squares using sterilized scissors. The negative control MUS was returned to the ethanol bath until it was washed with 5 mL of phosphate-buffered saline (PBS), to not degrade the fluorescence of the stains.

To form positive control MUS, a sterilized 1 × 1 cm square of PPM MUS was inoculated with laboratory strain *P. aeruginosa* (PA01). PA01 was streaked onto a Tryptone Soya Agar plate and then incubated at 37°C for 18 h. One colony was selected and transferred into 10 mL of Tryptone Soya Broth (TSB) for a further 48 h. The MUS was placed in a 2 mL TSB with 1:200 PA01 for static inoculation at 37°C for 24 h. Control MUS were placed in a sterile container with 5 mL formalin for fixation to replicate the storage conditions of explanted MUS.

### Staining and probes

#### TOTO-1

MUS was stained with TOTO-1 by submerging in a mixture of the stain with PBS (TOTO-1 2 μL, 1 mM stock, in 1 mL PBS) for 5 min, at 37°C, and protected from light.

#### Eubacterial FISH probe

The bacterial 16S rRNA-targeting probe EUB338 (5′-GCTGCCTCCCGTAGGAGT-3′) was used (25). This has previously been applied successfully to visualize bacterial cells amongst human tissue (26–28). This was a custom fluorescence probe, and it was necessary to ensure that it did not overlap with the wavelengths of other probes. Cy-3 was selected; this has been used in previous studies (27). Cy-3 emits an orange fluorescence, with an excitation of 555 nm and an emission of 569 nm.

#### Counterstaining

DAPI (4′,6-diamidino-2-phenylindole) was used as a nucleic-acid counterstain. It is excited at 341 nm and has a peak emission at 452 nm. All images were obtained using the confocal laser scanning microscopy (CLSM) settings described below.

#### Validated confocal laser scanning microscopy (CLSM) staining protocol

The MUS was removed from formalin, washed with PBS, and placed in a well with 50 μL hybridization buffer (20 mM Tris-HCl, 0.9 M NaCl, 0.1% [wt/vol] SDS), which had been prewarmed at 37°C for 15 min. Then, 0.5 μL of EUB338 FISH probe (1 pmol/μL) was added to the well, using an adaptation from previously described methodology (27) as the MUS was already fixed in formalin and so already partially prepared. The wells were protected from light and placed at 37°C for 2 h. The MUS was then washed with PBS and then submerged in 1 mL of TOTO-1 (2 μL, 1 mM stock) in PBS solution for 5 min at 37°C and protected from light. After washing with PBS, the MUS was submerged in 2 mL of PBS with DAPI (4 μL, 30 nM stock) at room temperature for 1 min. The MUS was washed with PBS three times, prior to submersion in a well of PBS for imaging.

### Validated CLSM settings

Images were collected with a Leica TCS SP8 AOBS inverted confocal using a 20× water immersion objective, with the stained mesh submerged in PBS. The confocal settings were as follows: pinhole one airy unit, scan speed 1,000 Hz unidirectional, format 1,024 × 1,024. Images were collected using hybrid detectors with the following detection mirror settings: DAPI 341–452 nm, TOTO-1 514–533 nm, and Cy-3 555–569 nm using the white light laser with 358 nm (10%), 514 nm (10%), and 555 nm (10%) laser lines, respectively. The images were collected sequentially. When acquiring 3D optical stacks, the confocal software was used to determine the optimal number of Z sections. Only the maximum intensity projections of these 3D stacks are shown in the results.

### Image rendering and quantification

Images were rendered in iMaris and probe-specific volumes were calculated from standardized thresholded volumes. Relative volumes were then calculated to allow comparison between samples by expressing each probe-derived volume as a proportion of the total rendered signal/volume for that sample. Settings for volume rendering were standardized to involve all voxels, and the minimum threshold or background subtraction was set between 20 and 30 per probe, dependent on the 25th percentile. The threshold is typically accepted to be an overestimate, and therefore the 25th percentile was used. Calculation of relative volumes using this software has been used in other studies to quantify biofilms (29–32).

### Statistical analysis

Statistical analysis was conducted using SPSS 25. Data were explored for distribution and outliers. Parametric data are reported as means with standard deviation and underwent analysis using paired *t*-test or one-way analysis of variance (ANOVA); non-parametric data are reported as medians with interquartile range and underwent analysis using Mann-Whitney *U* or Kruskal-Wallis. Groups for comparisons included complication groups, mesh location, MUS route, and subgroup analysis of vaginal mesh and mesh arms with complication site.

## RESULTS

### Background demographics

A total of 122 mesh samples underwent imaging for this study. Ten mesh samples were used for development and validation of the imaging methodology. The results reported are for 112 mesh samples, which underwent our validated imaging methodology; all these samples showed evidence of biofilms. These mesh samples belonged to a total of 52 women, some women provided multiple samples of mesh, and this was analyzed by anatomical location. Table S1 summarizes the background demographics of the women who participated in this study, and Table S2 summarizes previous treatment, location of mesh, and indication for excision.

### Confocal microscopy evidence of biofilms

**Fig 1:**
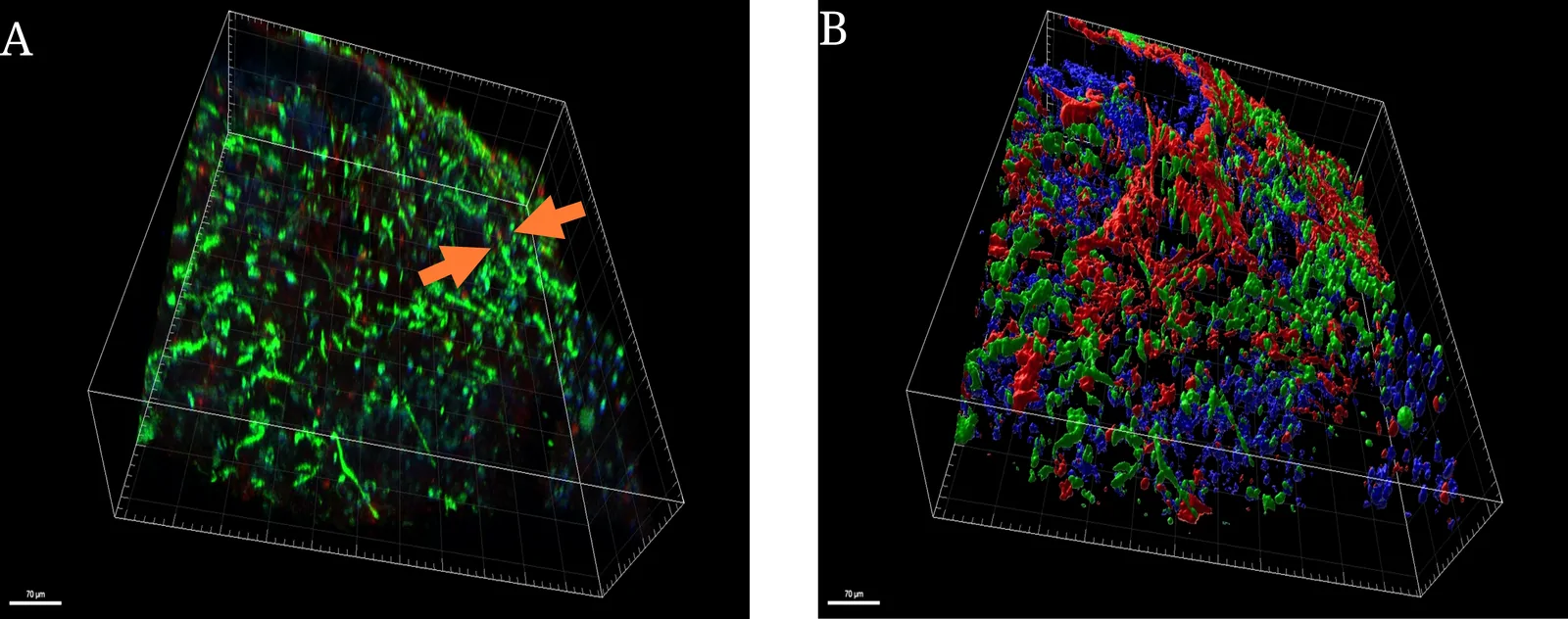
Vaginal portion of retropubic MUS from a woman with chronic pain. Scale bar = 70 µm. (**A**) 3D rendered image; (**B**) corresponding generated volume image. Red indicates EUB338 FISH signal, consistent with bacterial cells; green indicates TOTO-1 signal, consistent with extracellular nucleic acid/eDNA associated with biofilm-like material; and blue indicates DAPI nucleic-acid counterstain. DAPI was used as a general nucleic-acid counterstain and is not specific for host cells, as it can also bind bacterial DNA. However, larger blue-stained structures with nuclear morphology were interpreted as host-cell-associated material where present. Orange arrows indicate EUB338-positive bacterial cells associated with surrounding TOTO-1-positive biofilm-like material.

**Fig 2:**
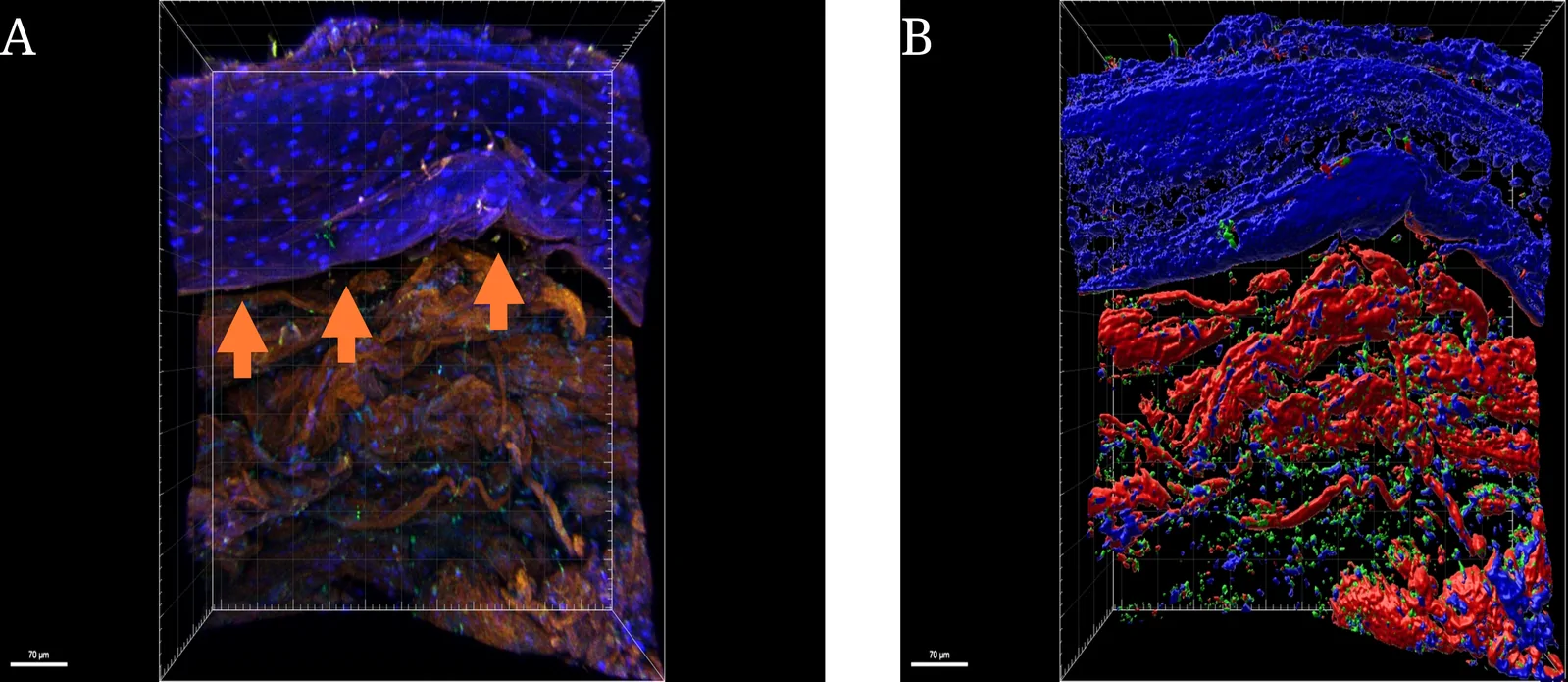
Vaginal portion of obturator MUS from a woman with chronic pain. Scale bar = 70 µm. (**A**) 3D rendered image; (**B**) corresponding generated volume image. Colors are as described in Fig. 1. Orange arrows indicate larger DAPI-positive structures with nuclear morphology, interpreted as host-cell-associated material, surrounded by EUB338-positive bacterial cells.

**Fig 3:**
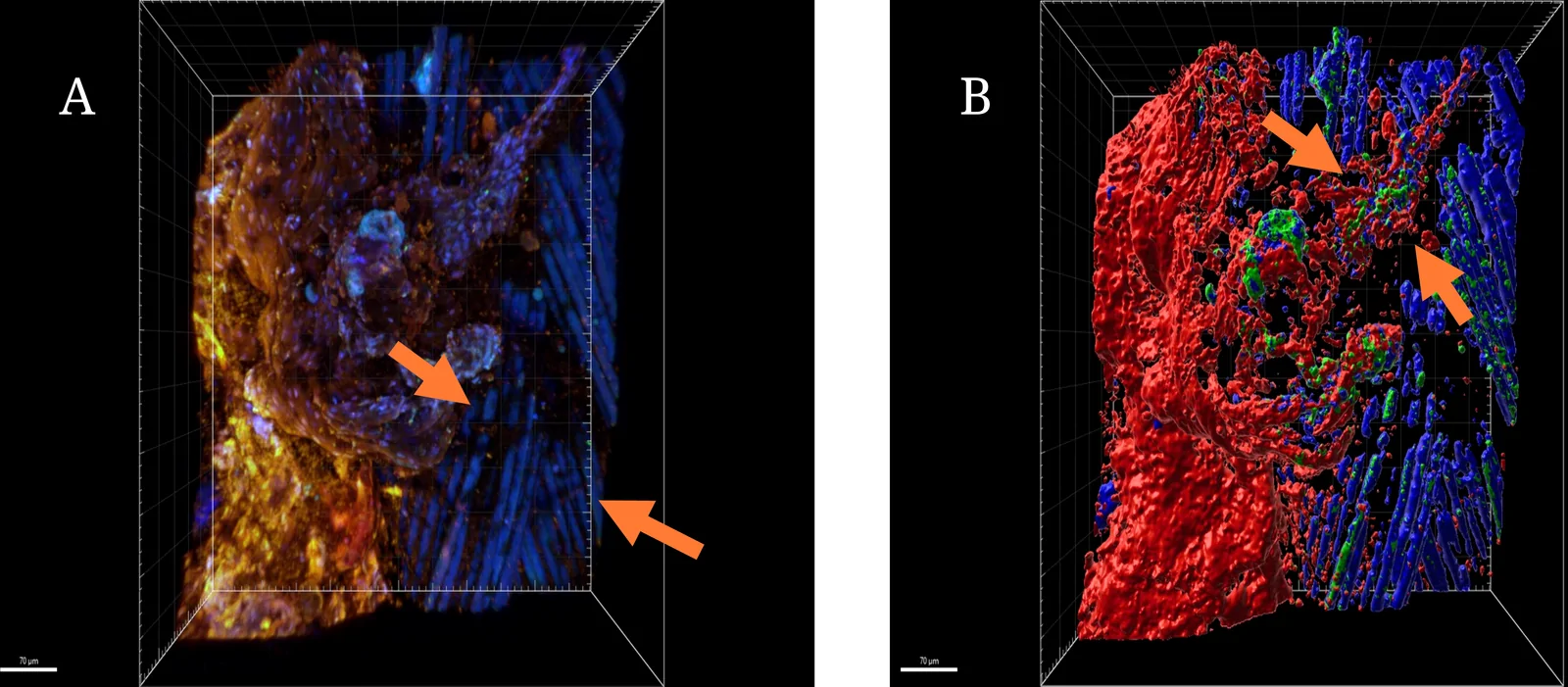
Vaginal portion of exposed retropubic MUS. Scale bar = 70 µm. (**A**) 3D rendered image; (**B**) corresponding generated volume image. Colors are as described in Fig. 1. Orange arrows indicate mesh-associated material within the exposed region, with EUB338-positive bacterial cells and surrounding TOTO-1-positive biofilm-like material more clearly visualized in the generated volume image in panel B.

**Fig 4:**
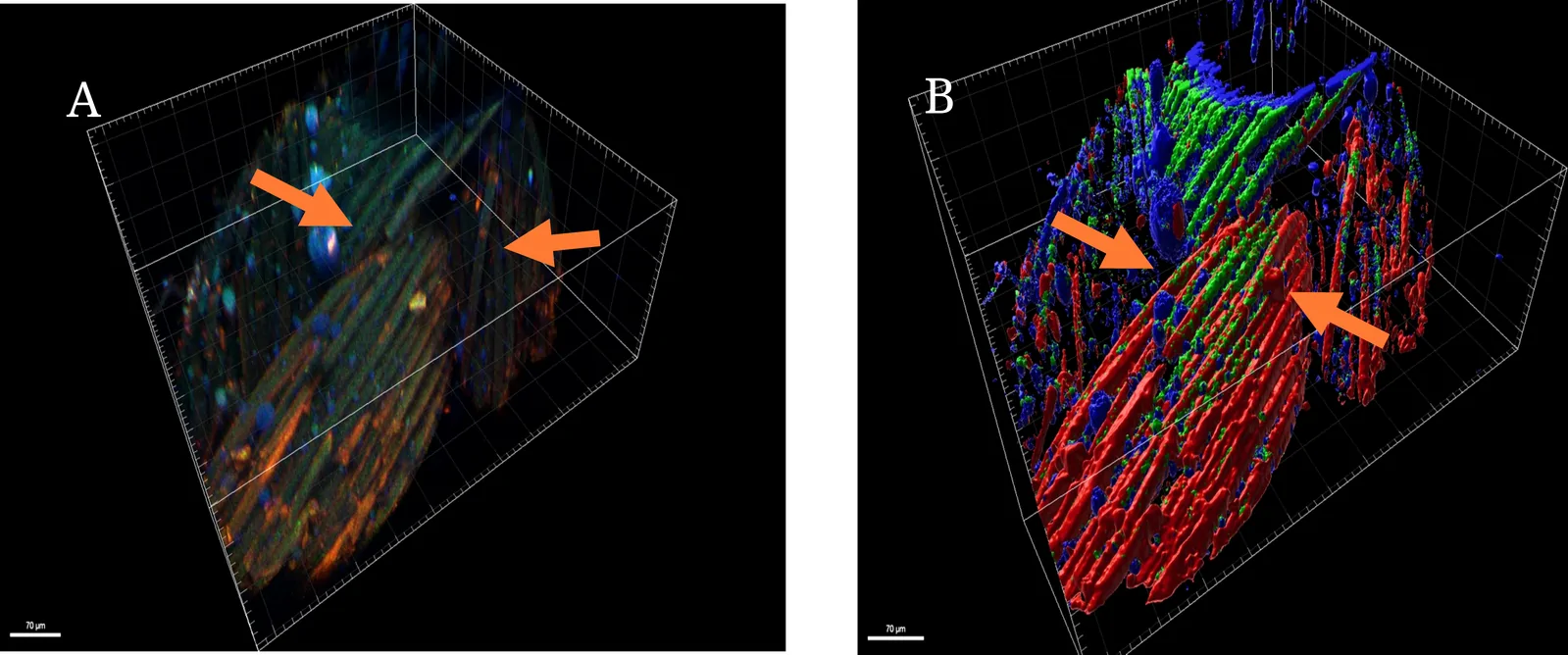
Vaginal portion of exposed obturator MUS. Scale bar = 70 µm. (**A**) 3D rendered image; (**B**) corresponding generated volume image. Colors are as described in Fig. 1. Orange arrows indicate mesh-associated material within the exposed region, with EUB338-positive bacterial cells and surrounding TOTO-1-positive biofilm-like material more clearly visualized in the generated volume image in panel B.

**Fig 5:**
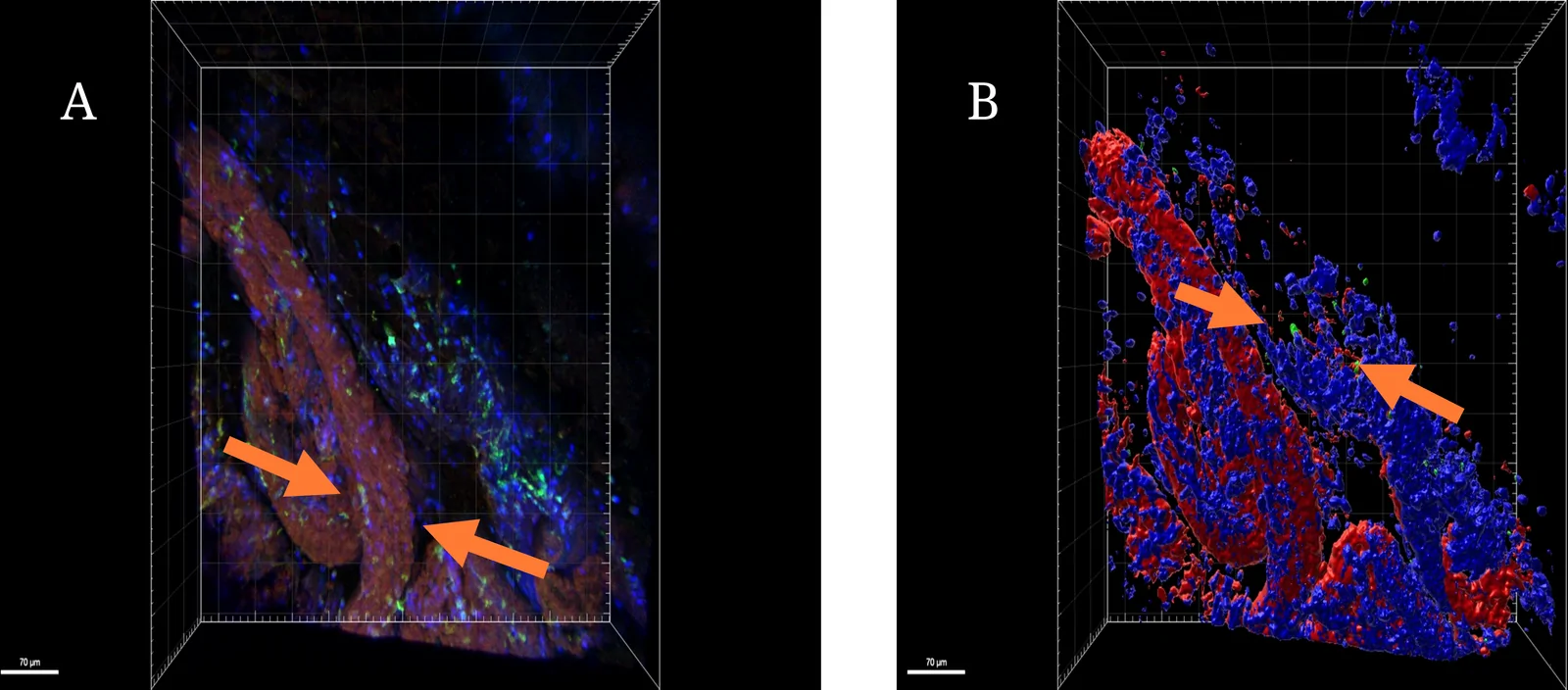
Vaginal portion of retropubic MUS that had perforated through the lower urinary tract. Scale bar = 70 µm. (**A**) 3D rendered image; (**B**) corresponding generated volume image. Colors are as described in Fig. 1. Orange arrows indicate EUB338-positive bacterial cells associated with surrounding TOTO-1-positive biofilm-like material.

**Fig 6:**
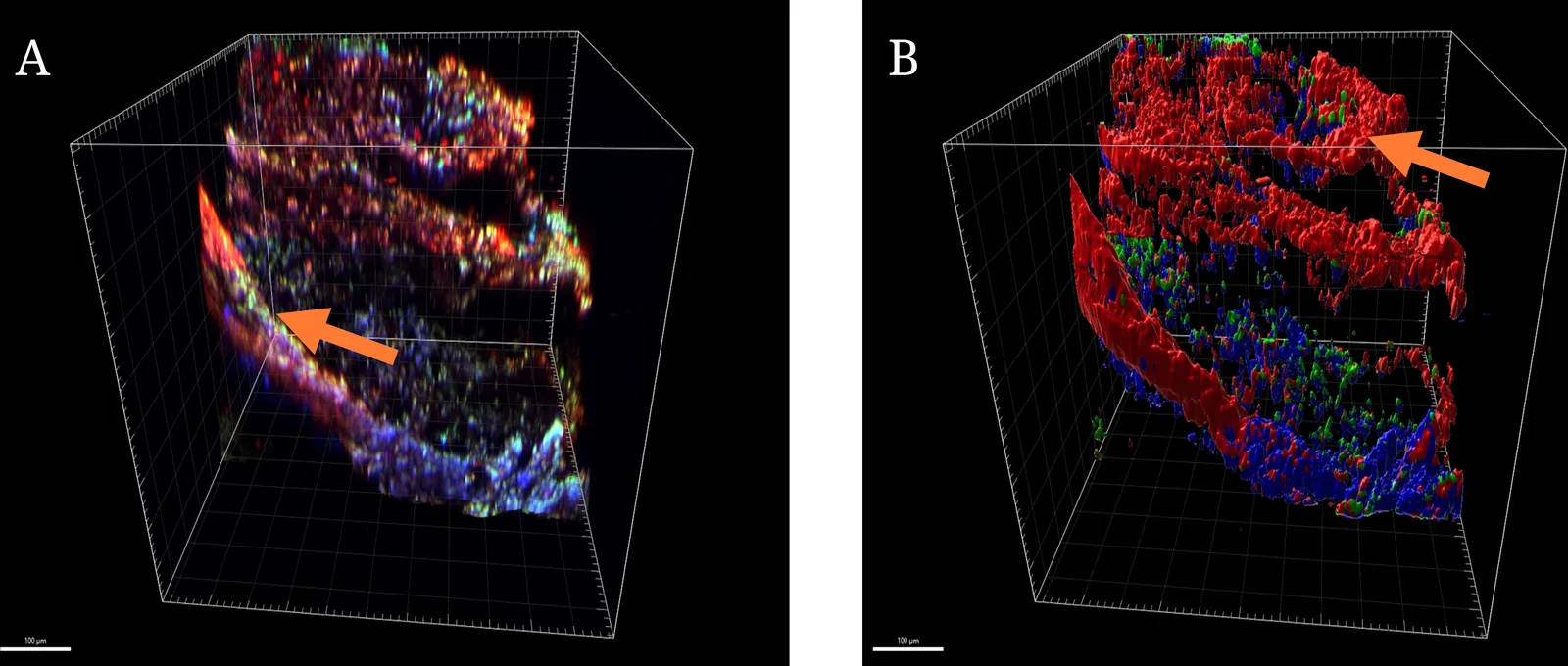
Vaginal portion of obturator MUS that had perforated through the lower urinary tract. Scale bar = 100 µm. (**A**) 3D rendered image; (**B**) corresponding generated volume image. Colors are as described in Fig. 1. Orange arrows indicate EUB338-positive bacterial cells associated with surrounding TOTO-1-positive biofilm-like material.

All samples showed widespread uptake of FISH EUB338 as cocci, rods, and spirals in association with nearby uptake of TOTO-1. This finding is highly suggestive of bacterial biofilms. These findings were replicated irrespective of anatomical location and complication. Figures 1 to 6 display a representative sample of 3D rendered images of vaginal mesh, from women with various complications, alongside the generated volumes. Figures S1 to S5 display representative images of retropubic or obturator arms of the MUS. Figure S6 is from the portion, which sits above the rectus sheath. These images show that although an image may appear to have a higher uptake of FISH or TOTO-1, when the projections are accounted for by volume rendering, this subjective interpretation by eye alone may not be accurate; the same is true for areas of lower uptake. Of interest, imaging of mesh exposed within the vagina did not demonstrate an increased bacterial burden at the site of exposure. This is illustrated in Fig. 3 and 4. The regions appearing blue, with rope-like morphology, correspond to the exposed mesh material.

### Comparison of relative probe volumes between complication groups

Probe uptake was expressed as a proportion of the total signal for each sample to enable comparisons between samples. Prior to comparing relative volumes between groups, normality of distribution was assessed using the Shapiro-Wilk test (*P* > 0.05), and data were screened for outliers. For the relative volume of FISH signal, one outlier was identified in the “Chronic Pain” group. For TOTO-1, one outlier was identified in the “Exposure” group. For DAPI, three outliers were identified in the “Chronic Pain” group and one in the “Control” group. As no consistent outlier was present across probes, and given the small sample size within each group, all data points were retained for further analysis.

**Fig 7:**
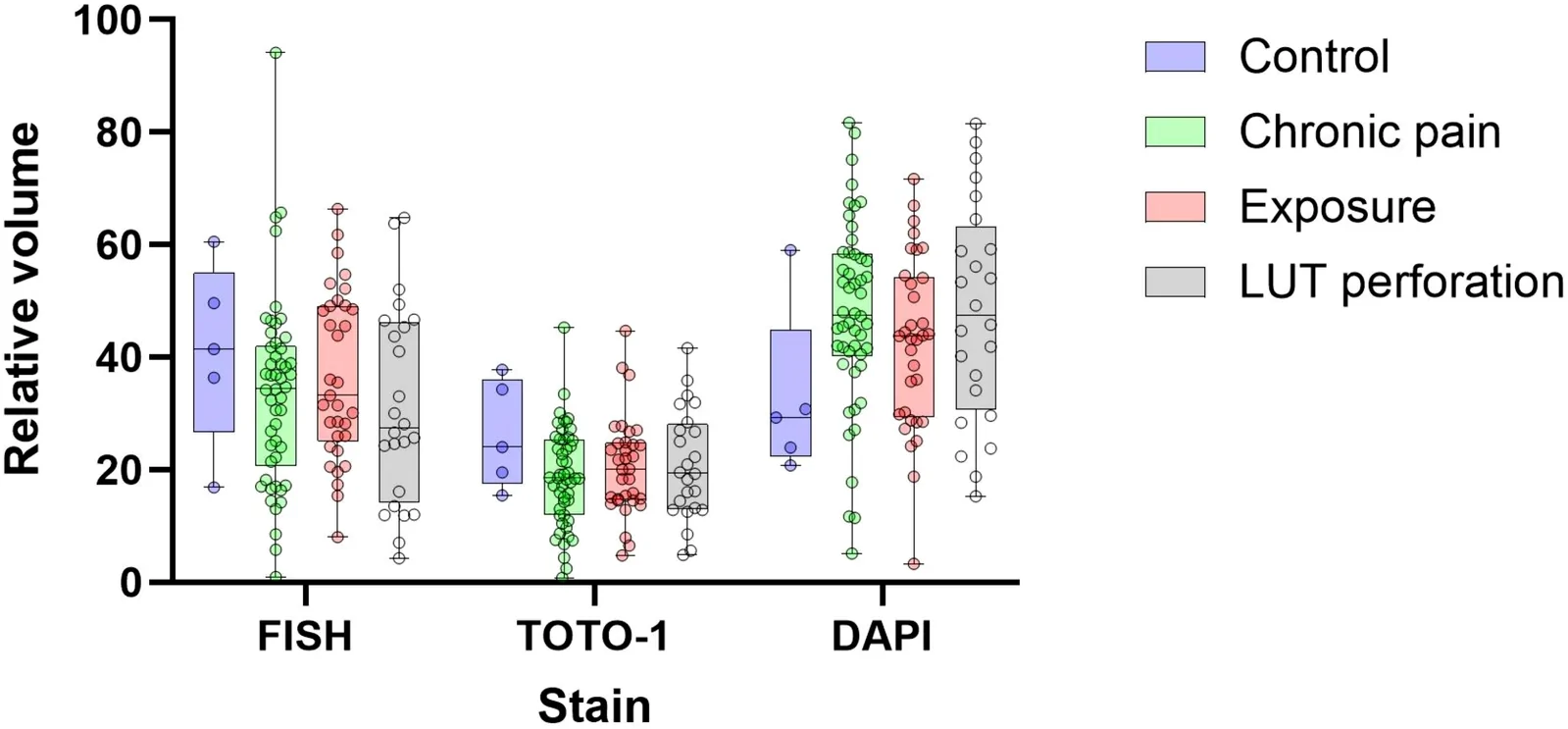
Mean relative volumes of FISH, TOTO-1, and DAPI, by complication group. No significant differences were found between complication groups (error bars = standard deviation) (*P* > 0.05) (Table S4).

One-way ANOVAs were performed to compare the mean relative volumes of FISH, TOTO-1, and DAPI signals between complication groups across all anatomical sites (Fig. 7). No significant differences were observed between groups (*P* > 0.05).

**Fig 8:**
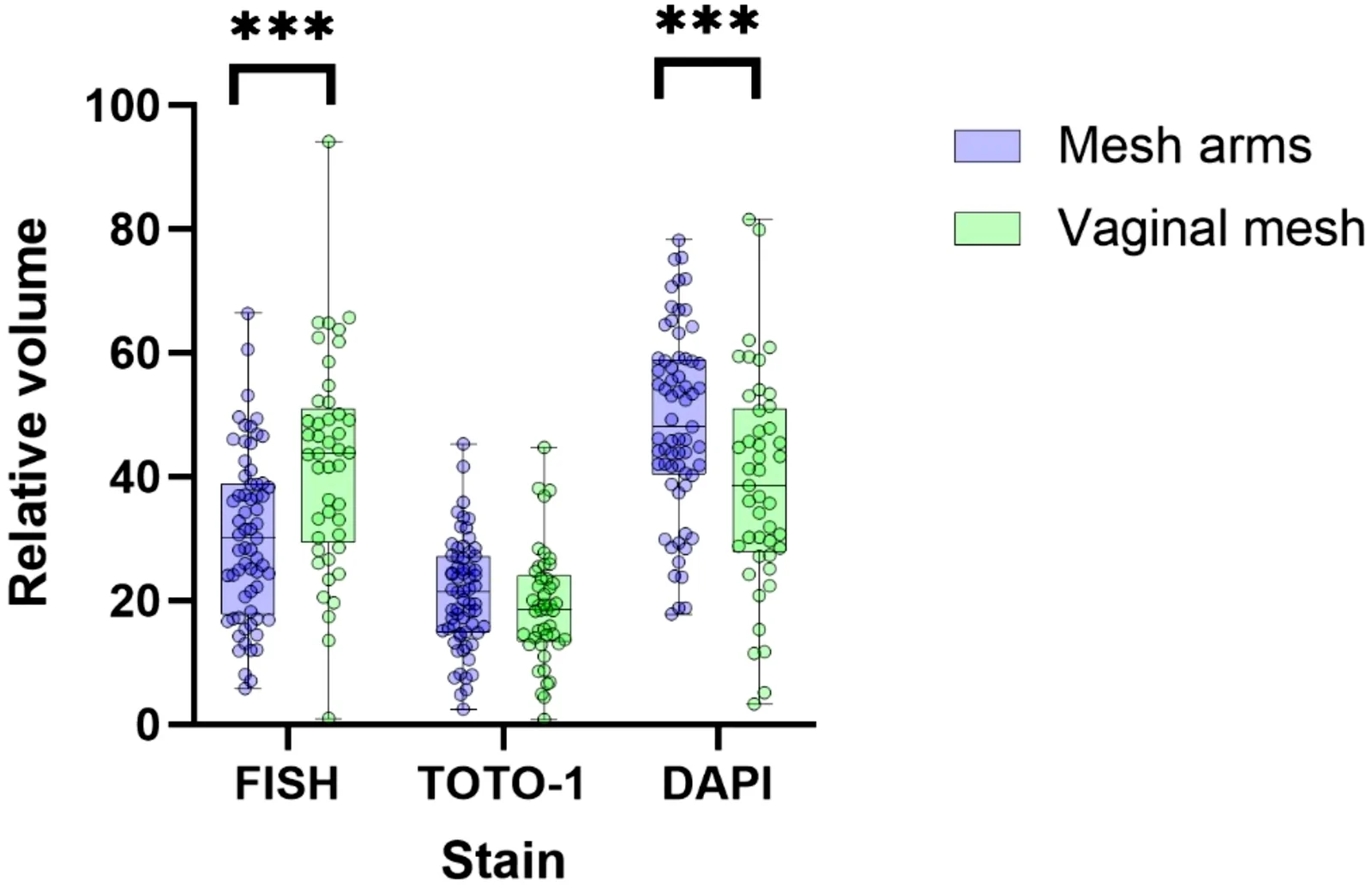
Relative volumes of FISH, TOTO-1, and DAPI, by whether the sample was mesh arms or vaginal mesh. There was significantly higher FISH relative volume in the vaginal mesh and significantly higher DAPI in the mesh arms (*P* ≤ 0.001). Significant finding noted by *** = *P* ≤ 0.001, *P*-value provided in Table S4.

Vaginal mesh (*n* = 45) was compared to mesh arms (*n* = 67); as relative volumes were normally distributed, independent-samples *t*-tests were conducted (Table S3) and represented in Fig. 8. There was a significantly higher volume of FISH in vaginal mesh (42 ± 17) compared to mesh arms (29.1 ± 13.8) (95% confidence interval [CI] = −18.7 to −7.1, *P <* 0.0005), and higher volume of DAPI in mesh arms (50.1 ± 15.5) compared to vaginal mesh (39 ± 17.4, 95% CI = 4.9 to 17.3, *P* = 0.001).

**Fig 9:**
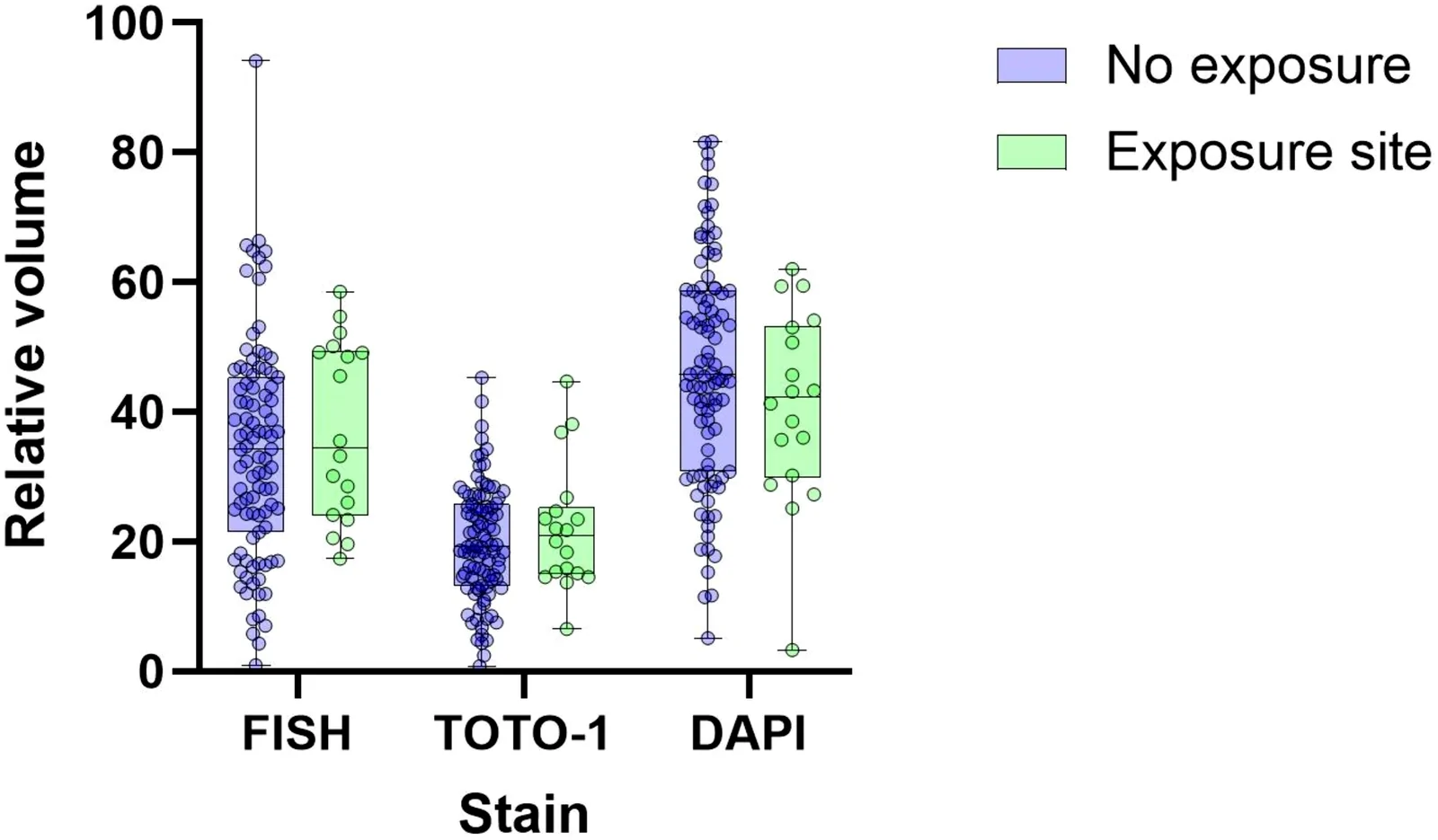
Mean relative volumes of FISH, TOTO-1, and DAPI in vaginal mesh samples only, comparing exposed mesh with covered mesh. There was no significant difference between no exposure and exposure site (*P* > 0.05).

Vaginal mesh samples were compared using independent-samples t-tests, as data were found to be normally distributed, with one outlier in the relative volume of TOTO-1 and one in the relative volume of FISH. Outliers were included due to the small sample sizes. No significant differences were found between relative volumes of any probe when compared by exposure site, pain site, or LUT perforation site (Table S4). Further analysis of vaginal mesh compares mesh, which was exposed in the vagina with mesh, which was covered; that is, LUT perforation group was excluded. No significant difference was found in biofilm volumes of the vaginal portion of mesh when comparing exposed with covered mesh, as seen in Fig. 9.

Relative volume data from mesh arms were found to have non-parametric distribution, and so further testing between groups was with Mann-Whitney *U* or Kruskal-Wallis if there were more than two groups.

There were no significant differences when assessing relative volumes by route (retropubic or obturator) of mesh, or site of pain, across all probes (*P* > 0.05) (Table S5).

## DISCUSSION

The widespread detection of bacterial cells by the bacterial-specific EUB338 FISH probe, revealing coccoid, rod-shaped, and spiral morphologies, together with associated TOTO-1 staining of extracellular nucleic acids, confirmed the presence of bacterial aggregates consistent with biofilms on all successfully imaged mesh samples. Although extracellular DNA may also arise from damaged or necrotic host cells, the close spatial association between TOTO-1 staining and bacterial FISH signal supports its interpretation as part of the biofilm matrix. These findings indicate that although MUS are implanted under surgical aseptic conditions, bacterial adherence occurs after vaginal insertion, allowing biofilm formation and evasion of host immune clearance. The detection of biofilms on MUS aligns with and strengthens previous observations suggesting that polypropylene mesh can serve as a substrate for bacterial colonization and biofilm development (15, 16) and current literature that reports the finding of biofilms on polypropylene (21, 22, 33, 34). Biofilms were not unique to women with mesh complications; they were also observed in the control group, although the number of control samples was small (five mesh samples from three women). These findings suggest that biofilms may form on all MUS and that their presence alone does not necessarily result in complications. It is important to note that the women in the control group were undergoing secondary stress incontinence surgery, so they may be considered to have a “failed” MUS; women with successful SUI treatment with a MUS and no complications may have yielded different results. It was not possible to include this cohort as women without complications and successful treatment do not typically undergo excision of their MUS, and no such woman underwent explantation surgery within the unit over the study period.

Biofilm biovolume did not differ between complication groups, suggesting that complication development may not be determined simply by the density of biofilm present. Instead, differences in biofilm composition may be relevant. Biofilm matrices vary between bacterial species and growth conditions (35) and may contain differing proportions of exopolysaccharides, proteins (36), and extracellular DNA (37). Importantly, experimental evidence indicates that variation in biofilm matrix constituents can alter host immune cell responses, including neutrophil activation. Thus, the microbial composition and matrix chemistry of the biofilm, rather than its biovolume alone, may influence downstream inflammatory responses and clinical complications (38).

There appears to be a greater bacterial burden on the vaginal portion of the mesh compared with the mesh arms. This likely reflects the anatomical location, as mesh residing within the vagina is exposed to a higher microbial load. In contrast, mesh positioned further from the vaginal environment would be expected to harbor less biofilm. Interestingly, vaginal mesh exposure was not associated with a higher volume of bacteria when specifically assessing vaginal mesh. This may suggest that constant exposure to the vaginal microbiota does not routinely result in a greater bacterial burden on the MUS. This is further supported by the finding that there was no evidence of an increased bacterial burden on mesh arms in the vaginal mesh exposure group. The portion of mesh, which itself was exposed, often visually appeared to carry less bacteria than the adjacent covered portion. This may be a result of the environment of the vagina itself, rather than when mesh is concealed by vaginal skin. It is possible that exposure to weak shear forces within the vagina results in removal or flattening of the biofilm. Alternatively, it may be related to the bacteria that are present within this complication group; these bacteria may form biofilms less readily. This study did not include women with mesh infection, and so another explanation may be that there is a threshold of bacterial burden, which leads to mesh infection rather than chronic colonization as we have seen in this cohort. This is supported by previous work that found low levels of bacterial quantification when culturing mesh, which was excised for complication (15, 16). This study did not find any measurable differences in biofilm volumes between complication and control groups or route of mesh. Biofilms were detected on all samples analyzed in this study, including samples from women with differing reported antibiotic, probiotic, or vaginal estrogen usage.

Biofilms have been implicated in many disease states and have been found to be resistant to treatment without excision when existing within the human body (39), especially on medical devices. Urinary catheters are relatively easy to replace—replacing these reduces the risk of urinary tract infections. Materials that reduce the risk of biofilm formation are being tested for catheters (40). In the case of breast implants, biofilms were found to be a factor in fibrosis and capsular contraction (41, 42). Capsular contracture is found to worsen over time, and surgery to excise the “capsule,” or biofilm, is considered the gold-standard treatment (43). Interestingly, ultrasound is being investigated as a tool for the detection of breast implant contracture (44, 45); this is an area that requires further research for MUS (46).

In the case of MUS, biofilms were observed on all samples, including from women without complications. Biofilm volume did not differ between complication groups, suggesting that biofilm burden alone is unlikely to determine the degree of inflammation or chronic pain. It is possible that certain biofilms result in a histological response, which promotes fibrosis and inflammation; these steps may provide a target for the prevention of complications.

As far as we are aware, this is the largest study assessing *ex vivo* biofilms on polypropylene mesh and the only one reporting biofilms on MUS; other studies are *in vivo* (21) and use hernia mesh from those with mesh infection (22) or vaginal prolapse mesh (23), and one has only been published as an abstract (34). In this study, we have provided evidence of biofilms on excised polypropylene MUS.

A recent study (23) imaged bacterial biofilms on two vaginally explanted mesh samples used for prolapse surgery. Standard block and sectioning techniques were used, and bacterial DNA was labeled with a eubacterial FISH probe; other specific biofilm constituents were not targeted. This approach was similar to that used in several other studies of biofilms on mesh. Our study differed by aiming to detect extracellular DNA (eDNA), a biofilm constituent. However, TOTO-1 may also detect extracellular DNA released from host cells damaged during surgery. Due to the formalin fixation of cells followed by permeabilization for FISH staining, it is possible that some of the eDNA probe (TOTO-1) would enter cells and become less specific in labeling only eDNA in biofilms. The detection of TOTO-1 alongside clusters of bacterial DNA (using the eubacterial FISH probe) provides a more robust methodology for the detection of biofilms than described in previous studies.

The widespread detection of EUB338-positive bacterial cells, revealing coccoid, rod-shaped, and spiral morphologies, together with associated TOTO-1 staining of extracellular nucleic acids, supported the presence of biofilm-like bacterial aggregates on all successfully imaged mesh samples. Although extracellular nucleic acids may also derive from damaged or necrotic host cells, the close spatial association of TOTO-1 signal with bacterial FISH-positive structures was consistent with an extracellular biofilm matrix.

Alternative matrix stains could provide additional information on biofilm composition. For example, protein- or polysaccharide-targeted stains may help distinguish different extracellular matrix components. However, in explanted vaginally implanted material, such stains may also label host-derived material, including cellular debris or host extracellular matrix. We therefore interpreted TOTO-1 staining cautiously and in relation to its spatial association with EUB338-positive bacterial cells, rather than as a standalone marker of biofilm.

A limitation of the study is that we were not able to label specific bacteria and get compositional data using FISH staining. Labeling specific bacteria may help to further characterize biofilms and identify differences between mesh complications. This, paired with histological analysis, may provide further insights into the host response to different biofilms and the pathophysiology of mesh. Furthermore, follow-up of pain outcomes of women should be associated with biofilm volume, as this was not assessed in this study but may find further evidence for the role of mesh excision in the management of chronic pain associated with MUS.

### Conclusion

Bacterial biofilms were observed on all MUS, irrespective of complication or control group and anatomical location. This study demonstrates that bacteria can persist as structured biofilms on polypropylene MUS. There were no significant differences between complication and control groups in the volume of biofilms, suggesting that biofilm burden alone is unlikely to be the primary driver of MUS complications. Biofilm composition, including the bacterial species present and the composition of the EPS matrix, may influence chronic inflammation. The vaginal portions of the mesh exhibited a significantly higher bacterial burden, consistent with a greater biofilm burden compared with the retropubic or obturator mesh arms. This likely reflects their anatomical proximity to the vagina, which harbors a dynamic resident microbiome. However, vaginal mesh exposure itself was not associated with a significant increase in biofilm volume on the vaginal mesh. This raises the possibility of a threshold between chronic colonization with biofilms and mesh infection, a cohort that was not included in our study, as the cause of this complication is more clearly microbial.

Further research will aim to characterize differences in the composition of bacterial biofilms and determine whether these are associated with mesh complications. This will include further investigation of the mesh microbiome and the local microbial environment. Strategies to prevent and treat implant-associated biofilms are an active area of research; if biofilms are shown to contribute to MUS complications, approaches to reduce biofilm formation may have potential clinical value. For those with a MUS, further investigation of the role of ultrasound in the diagnosis of MUS complications may help guide further management if biofilms may be detected.

## SUPPLEMENTAL MATERIAL

Supplemental material aem.00735-26-s0001.pdf Tables S1 to S5; Fig. S1 to S6.

ASM does not own the copyrights to Supplemental Material that may be linked to, or accessed through, an article. The authors have granted ASM a non-exclusive, world-wide license to publish the Supplemental Material files. Please contact the corresponding author directly for reuse.
