# Supplementary material for: Detection of biofilms on explanted urinary incontinence slings and their relationship with pain, exposure, or urinary tract complications
Source: Appl Environ Microbiol. 2026 Aug 13;92(9):e00735-26. doi: 10.1128/aem.00735-26 (PMC13599481; doi:10.1128/aem.00735-26)
Supplement: Supplementary file 1 [file aem.00735-26-s0001.pdf]

**Table S1.** Background demographics of participants (n=52).

| | Average | SD or IQR |
|---|---|---|
| **Age** | 52.9 | 9.69 |
| **BMI** | 30.7 | 4.995 |
| **Years since index surgery** | 9.5 | 6.25 - 12 |
| **PainDETECT score** | 16<br>(n = 44) | 6.75 - 23.0 |
| | **n** | **%** |
| **Smokers (ex or current)** | 14 | 26.9 |
| **Diabetes** | 2 | 3.8 |
| **Autoimmune** | 5 | 9.61 |
| **Obturator** | 23 | 44.2 |
| **Retropubic** | 29 | 55.8 |
| **Presenting Symptoms (self-reported)** | | |
| **Pain** | 46 | 88.5 |
| **Dyspareunia** | 31 | 59.6 |
| **Vaginal bleeding** | 17 | 32.7 |
| **Vaginal discharge** | 13 | 25 |
| **Recurrent UTIs** | 26 | 50 |
| **Urgency** | 36 | 69.2 |
| **Recurrent SUI** | 38 | 73.1 |
| **Voiding Dysfunction** | 28 | 53.8 |

**Table S2.** Previous treatment, indication for surgery and location of sample.

| | n | % |
|---|---|---|
| **Previous treatment** | | |
| **Vaginal Oestrogen** | 6 | 11.5 |

| | | |
|---|---|---|
| **Probiotics** | 11 | 21.2 |
| **Antibiotics** | 13 | 25 |
| **Previous excision** | 12 | 23.1 |
| **Indication for surgery (n=52)** | | |
| **Control (recurrent SUI)** | 3 | 5.8 |
| **Chronic pain** | 23 | 44.2 |
| **Vaginal exposure** | 17 | 32.7 |
| **Lower urinary tract perforation** | 9 | 17.3 |
| **Bladder** | 1 | 11.1 |
| **Urethral** | 8 | 88.9 |
| **Location of Mesh (n = 112)** | | |
| **Vaginal** | 45 | 40.2 |
| ***Exposure*** | 17 | 37.8 |
| ***LUT perforation*** | 10 | 22.2 |
| ***Not exposed or in LUT*** | 18 | 40 |
| **Arms** | 64 | 57.1 |
| ***Obturator*** | 30 | 46.9 |
| ***Retropubic*** | 34 | 53.1 |
| **Rectus sheath** | 3 | 2.7 |

**Table S3.** Comparison of vaginal mesh with mesh arms, volumes of probes.

| | | | | | | | **95% Confidence Interval of the Difference** | |
|---|---|---|---|---|---|---|---|---|
| **Probe (Relative Volume)** | Location | N | Mean | SD | Std. Error Mean | p value | Lower | Upper |

| | | | | | | | | |
|---|---|---|---|---|---|---|---|---|
| **FISH** | Mesh arms | 67 | 29.1 | 13.8 | 1.7 | 0.0 | -18.7 | -7.1 |
| | Vaginal mesh | 45 | 42.0 | 17.0 | 2.5 | | | |
| **TOTO-1** | Mesh arms | 67 | 20.8 | 8.9 | 1.1 | 0.3 | -1.7 | 5.2 |
| | Vaginal mesh | 45 | 19.0 | 9.2 | 1.4 | | | |
| **DAPI** | Mesh arms | 67 | 50.1 | 15.5 | 1.9 | 0.0 | 4.9 | 17.3 |
| | Vaginal mesh | 45 | 39.0 | 17.4 | 2.6 | | | |

**Table S4.** Comparison of relative volumes of each probe of vaginal mesh, by pain site, exposure site and LUT perforation site.

| | | **N** | **Mean** | **SD** | **Standard Error Mean** | **p value (2 tailed)** | **95% Confidence Interval** | |
|---|---|---|---|---|---|---|---|---|
| **Relative volume** | | | **Pain site** | | | | Lower | Upper |
| **FISH** | No | 10.0 | 37.2 | 16.2 | 5.1 | 0.3 | -18.5 | 6.1 |
| | Yes | 35.0 | 43.4 | 17.2 | 2.9 | | -18.7 | 6.3 |
| **TOTO-1** | No | 10.0 | 19.7 | 8.9 | 2.8 | 0.8 | -5.8 | 7.7 |
| | Yes | 35.0 | 18.8 | 9.5 | 1.6 | | -5.9 | 7.8 |
| **DAPI** | No | 10.0 | 43.1 | 21.4 | 6.8 | 0.4 | -7.4 | 17.9 |
| | Yes | 35.0 | 37.8 | 16.3 | 2.8 | | -10.6 | 21.2 |
| | | | | **Exposure site** | | | | |
| **FISH** | No | 28.0 | 44.5 | 18.5 | 3.5 | 0.2 | -3.7 | 17.2 |

| | | | | | | | | |
|---|---|---|---|---|---|---|---|---|
| | Yes | 17.0 | 37.8 | 13.9 | 3.4 | | -3.0 | 16.5 |
| **TOTO-1** | No | 28.0 | 17.2 | 8.4 | 1.6 | 0.1 | -10.4 | 0.8 |
| | Yes | 17.0 | 22.0 | 10.0 | 2.4 | | -10.7 | 1.1 |
| **DAPI** | No | 28.0 | 38.3 | 19.0 | 3.6 | 0.7 | -12.9 | 9.0 |
| | Yes | 17.0 | 40.2 | 14.9 | 3.6 | | -12.2 | 8.4 |
| | | | | **LUT perforation site** | | | | |
| **FISH** | No | 35.0 | 42.1 | 17.3 | 2.9 | 0.9 | -11.8 | 13.1 |
| | Yes | 10.0 | 41.5 | 16.9 | 5.4 | | -12.4 | 13.6 |
| **TOTO-1** | No | 35.0 | 20.1 | 9.9 | 1.7 | 0.2 | -2.0 | 11.3 |
| | Yes | 10.0 | 15.4 | 5.7 | 1.8 | | -0.4 | 9.7 |
| **DAPI** | No | 35.0 | 37.8 | 16.9 | 2.9 | 0.4 | -17.9 | 7.4 |
| | Yes | 10.0 | 43.1 | 19.6 | 6.2 | | -20.0 | 9.5 |

**Table S5.** Comparison of relative volumes of probes of mesh arms.

| **Probe (Relative volume)** | | **N** | **Median** | **p- value** |
|---|---|---|---|---|
| | | **Mesh Route** | | |
| **FISH** | Obturator | 30 | 30.7 | 0.619 |
| | Retropubic | 34 | 28.3 | |
| **TOTO-1** | Obturator | 30 | 19.8 | 0.83 |
| | Retropubic | 34 | 21.7 | |
| **DAPI** | Obturator | 30 | 50.8 | 0.638 |
| | Retropubic | 34 | 47.1 | |
| | | **Pain site** | | |
| **FISH** | No | 29 | 28.5 | 0.594 |

| | | | | |
|---|---|---|---|---|
| | Yes | 35 | 32.3 | |
| **TOTO-1** | No | 29 | 18.3 | 0.399 |
| | Yes | 35 | 21.5 | |
| **DAPI** | No | 29 | 45.9 | 0.973 |
| | Yes | 35 | 52.3 | |
| | | **Complication group** | | |
| **FISH** | Chronic Pain | 33 | 32.4 | 0.278 |
| | Control | 4 | 43 | |
| | Exposure | 14 | 30 | |
| | LUT Perforation | 13 | 24.9 | |
| **TOTO-1** | Chronic Pain | 33 | 18.7 | 0.405 |
| | Control | 4 | 21.8 | |
| | Exposure | 14 | 20.9 | |
| | LUT Perforation | 13 | 26.8 | |
| **DAPI** | Chronic Pain | 33 | 53.1 | 0.465 |
| | Control | 4 | 30.1 | |
| | Exposure | 14 | 45.3 | |
| | LUT Perforation | 13 | 49.2 | |

**Supplementary Figures**

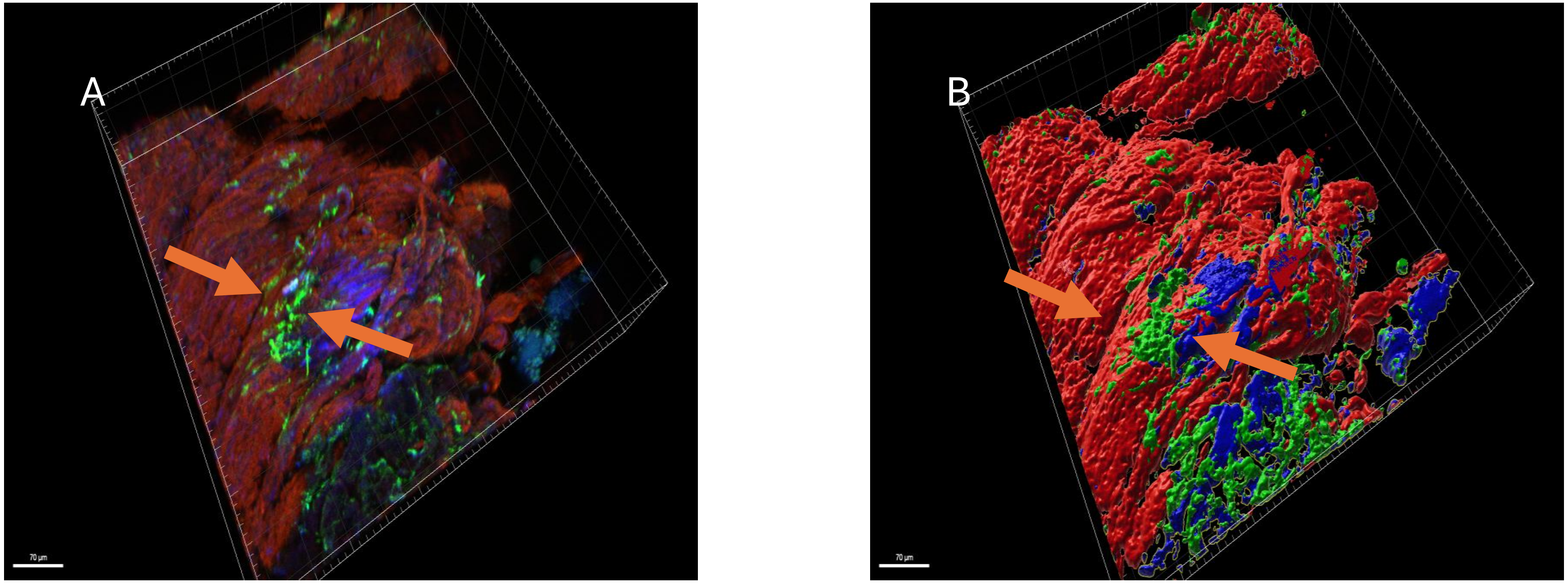


**Figure S1**. Mesh arms of retropubic MUS from a control participant, scale shown: 70µmA: 3D rendered images, and B:  corresponding generated volume image. Red = EUB338 FISH (bacteria), Green = TOTO-1 (eDNA), Blue = DAPI (generic nucleic counter stain suggesting host cells). Orange arrows show uptake by bacterial cells (red), surrounded by biofilms (green).

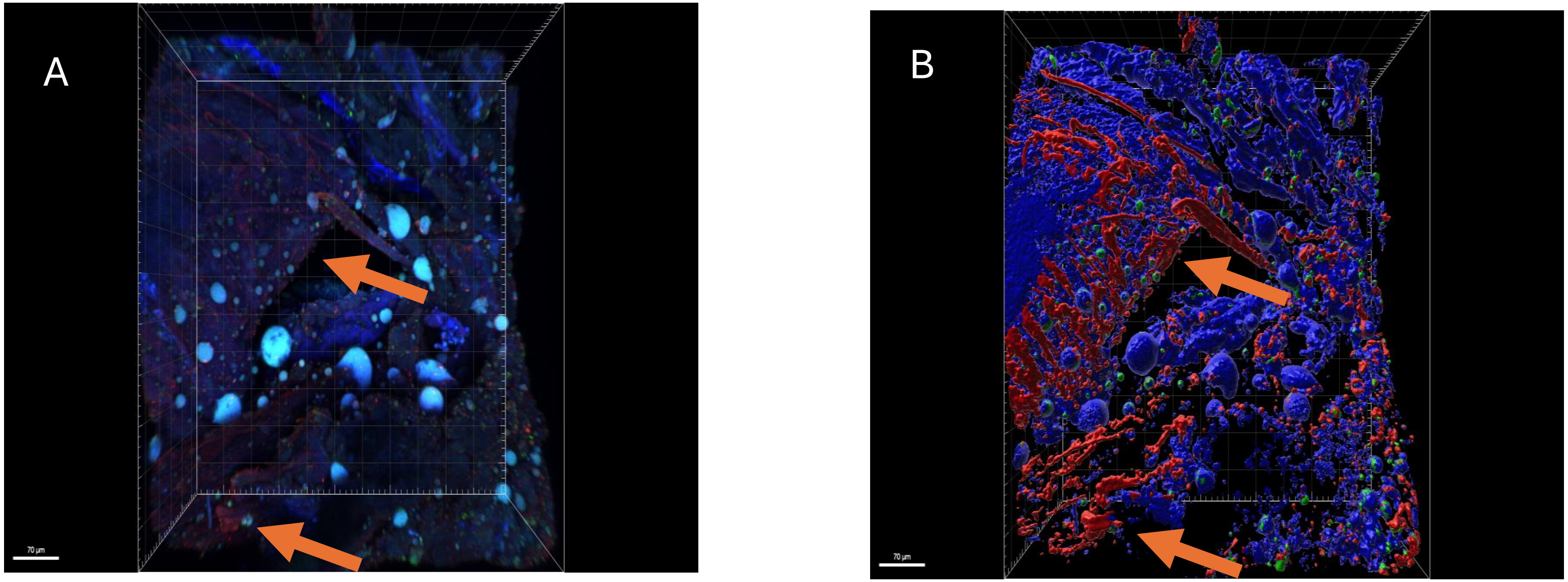


**Figure S2.** Mesh arms from an obturator MUS at <u>pain</u> site, scale shown: 70µm. A: 3D rendered images, and B: corresponding generated volume image. Red = EUB338 FISH (bacteria), Green = TOTO-1 (eDNA), Blue = DAPI (generic nucleic counter stain suggesting host cells). Orange arrows show uptake by bacterial cells (red), surrounded by biofilms (green).

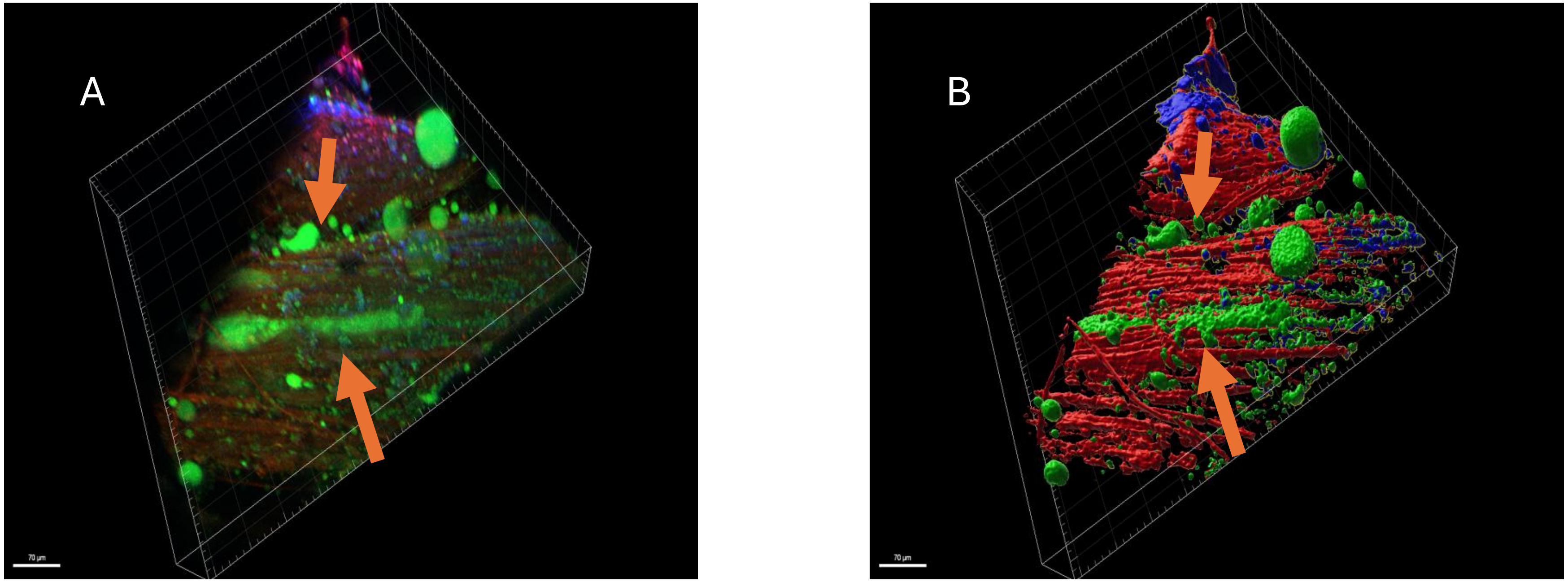


**Figure S3**. Mesh arms from a retropubic MUS at a pain site, scale shown: 70µm. A: 3D rendered images, and B: corresponding generated volume image. Red = EUB338 FISH (bacteria), Green = TOTO-1 (eDNA), Blue = DAPI (generic nucleic counter stain suggesting host cells). Orange arrows show uptake by bacterial cells (red), surrounded by biofilms (green).

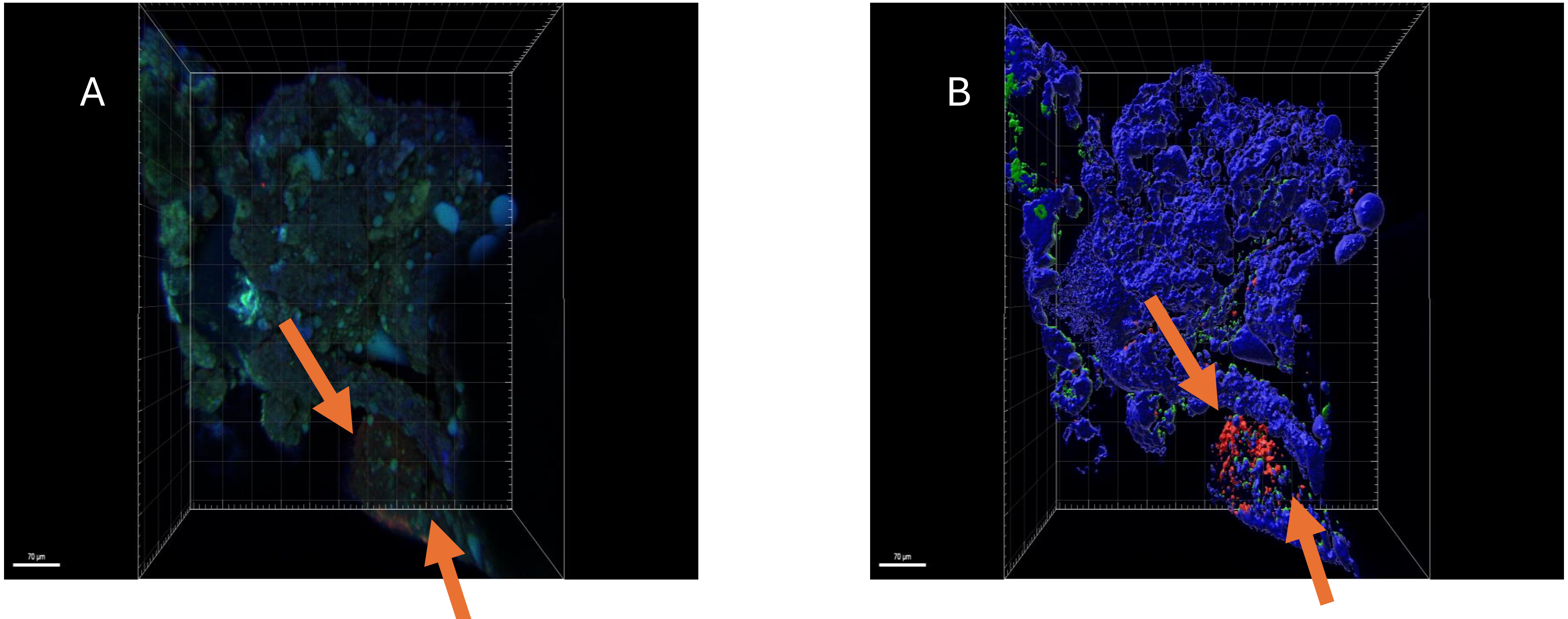


**Figure S4.** Mesh arms from a retropubic MUS with no pain at that site, scale shown: 70µm. A: 3D rendered images, and B: corresponding generated volume image. Red = EUB338 FISH (bacteria), Green = TOTO-1 (eDNA), Blue = DAPI (generic nucleic counter stain suggesting host cells). Orange arrows show uptake by bacterial cells (red), surrounded by little evidence of biofilms (green).

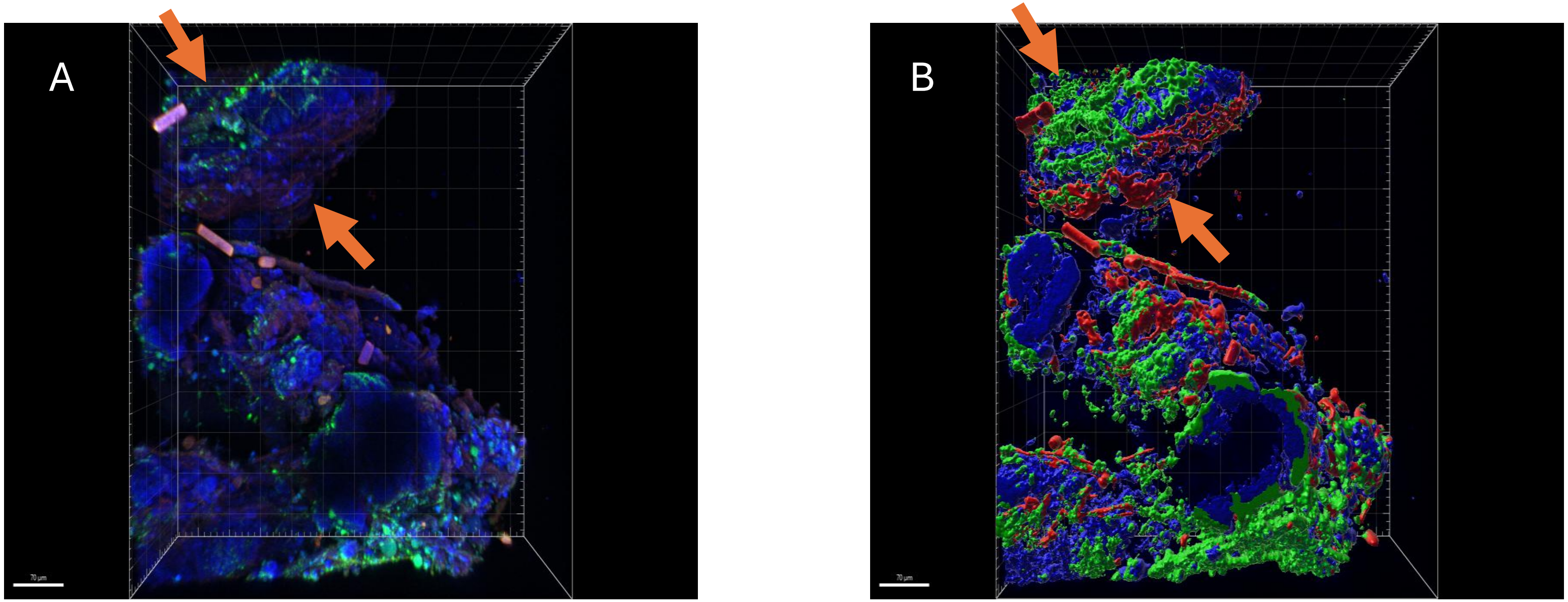


**Figure S5.** Mesh arms from an obturator MUS with no pain at that site, scale shown: 70µm MUS. A: 3D rendered images, and B: corresponding generated volume image. Red = EUB338 FISH (bacteria), Green = TOTO-1 (eDNA), Blue = DAPI (generic nucleic counter stain suggesting host cells). Orange arrows show uptake by bacterial cells (red), surrounded by little evidence of biofilms (green).

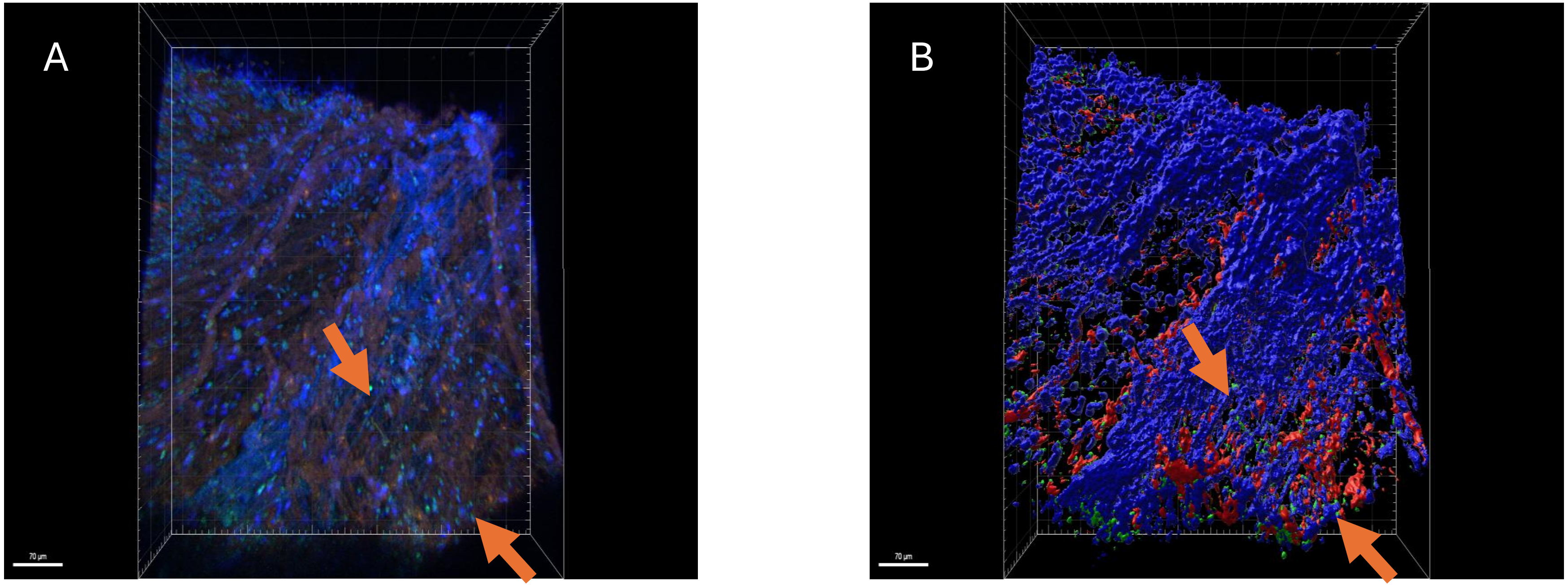


**Figure S6.** Mesh from the rectus sheath of a retropubic MUS, from a woman with chronic pain. A: 3D rendered images, and B: corresponding generated volume image. Red = EUB338 FISH (bacteria), Green = TOTO-1 (eDNA), Blue = DAPI (generic nucleic counter stain suggesting host cells). Orange arrows show uptake by bacterial cells (red), surrounded by little evidence of biofilms (green).
